# Chinese red yeast rice (*Monascus purpureus*) for primary hyperlipidemia: a meta-analysis of randomized controlled trials

**DOI:** 10.1186/1749-8546-1-4

**Published:** 2006-11-23

**Authors:** Jianping Liu, Jing Zhang, Yi Shi, Sameline Grimsgaard, Terje Alraek, Vinjar Fønnebø

**Affiliations:** 1 National Research Centre in Complementary and Alternative Medicine (NAFKAM), University of Tromso, Tromso N-9037, Norway; 2 Evidence-Based Medicine Centre in Traditional Chinese Medicine, Shanghai University of Traditional Chinese Medicine, Cai Lun Lu 1200, Pudong, Shanghai 201203, China; 3 The Institute of Traditional Chinese Medicine Literature, Shanghai University of Traditional Chinese Medicine, Cai Lun Lu 1200, Pudong, Shanghai 201203, China; 4 Evidence-Based Chinese Medicine Research Centre, Beijing University of Chinese Medicine, Beijing 100029, China

## Abstract

Extracts of Chinese red yeast rice (RYR, a traditional dietary seasoning of *Monascus purpureus*) contains several active ingredients including lovastatin, and several trials of its possible lipid-lowering effects have been conducted. This meta-analysis assesses the effectiveness and safety of RYR preparations on lipid modification in primary hyperlipidemia. We included randomized controlled trials testing RYR preparation, compared with placebo, no treatment, statins, or other active lipid-lowering agents in people with hyperlipidemia through searching PubMed, CBMdisk, TCMLARS, the Cochrane Library, and AMED up to December 2004. Ninety-three randomized trials (9625 participants) were included and three RYR preparations (Cholestin, *Xuezhikang *and *Zhibituo*) were tested. The methodological quality of trial reports was generally low in terms of generation of the allocation sequence, allocation concealment, blinding, and intention-to-treat. The combined results showed significant reduction of serum total cholesterol levels (weighted mean difference -0.91 mmol/L, 95% confidence interval -1.12 to -0.71), triglycerides levels (-0.41 mmol/L, -0.6 to -0.22), and LDL-cholesterol levels (-0.73 mmol/L, -1.02 to -0.043), and increase of HDL-cholesterol levels (0.15 mmol/L, 0.09 to 0.22) by RYR treatment compared with placebo. The lipid modification effects appeared to be similar to pravastatin, simvastatin, lovastatin, atorvastatin, or fluvastatin. Compared with non-statin lipid lowering agents, RYR preparations appeared superior to nicotinate and fish oils, but equal to or less effective than fenofibrate and gemfibrozil. No significant difference in lipid profile was found between *Xuezhikang *and *Zhibituo*. RYR preparations were associated with non-serious adverse effects such as dizziness and gastrointestinal discomfort. Current evidence shows short-term beneficial effects of RYR preparations on lipid modification. More rigorous trials are needed, and long-term effects and safety should be investigated if RYR preparations are to be recommended as one of the alternative treatments for primary hyperlipidemia.

## Background

Red yeast rice (RYR) is a traditional Chinese cuisine and medicinal agent prepared by using *Monascus purpureus *fermented with rice, which has been recorded in ancient Chinese pharmacopoeia Ben Cao Gang Mu-Dan Shi Bu Yi during the Ming Dynasty (1368–1644)[1]. The extracts from RYR contain starch, sterols, isoflavones, and monounsaturated fatty acids, and other compounds [2,3]; depending on *Monascus *strains used and fermentation conditions, it may contain polyketides called monacolins [4]. Monacolin K is lovastatin, which is a commonly prescribed lipid-lowering drug. Several randomized clinical trials have indicated beneficial effects of the RYR preparations including *Xuezhikang *and *Zhibituo *in the treatment of hyperlipidemia [5-9]. *Xuezhikang *has been in clinical use as a Chinese proprietary medicine in China and has recently been marketed in several European countries including Norway and Italy. As these preparations contain different compositions and concentration of lovastatin, evaluation of their effectiveness and safety from clinical trials is warranted.

People with hyperlipidemia have responded well to the lipid-lowering agents including HMG-CoA reductase inhibitors (statins), fibrates, nicotinic acids, and n-3 fatty acids [10]. However, long-term safety and potential drug interaction between statins and other hypolipidemic agents may become problematic [11-13]. Nowadays, many people would like to use naturaceuticals instead of chemical drugs. A previous systematic review identified four randomized trials of the lipid-lowering effects of RYR and concluded a lack of sufficient clinical research to support their efficacy [14]. The objective of this review is to assess the beneficial effects of lipid modification and safety of RYR preparations for their use in people with primary hyperlipidemia.

## Methods

### Search strategy

To identify relevant studies, we searched the following databases up to December 2004: The Cochrane Library, PubMed, Chinese Biomedical Database (CBMdisk), Traditional Chinese Medical Literature Analysis and Retrieval System (TCMLARS), and the Allied and Complementary Medicine Database (AMED). We used the search terms 'red yeast rice, *Monascus purpureus*, *Xuezhikang*, Cholestin, Hypochol, Hypocol, Lipascor', combined with 'hyperlipidemia, hypercholesterolemia, dyslipidemia, hypertriglyceridemia, hyperlipoproteinemia', and limited our search to clinical trials. Depending on the database, various combinations of both MeSH terms and the free text terms were used, but no limitation with regard to language and report type. We also screened the reference lists of identified papers and review articles, and contacted pharmaceutical companies.

### Inclusion criteria

We included randomized clinical trials comparing RYR vs. placebo, no intervention, or established lipid-lowering agents in individuals with primary hyperlipidemia on outcomes of lipid profile and adverse effects. Eligible trials had to include adult participants meeting the National Cholesterol Education Programme diagnostic criteria of hyperlipidemia [15] and excluded secondary causes such as hypothyroidism, familial hypercholesterolemia, diabetes mellitus, liver or kidney diseases. Trials comparing different RYR preparations were included, but trials comparing different dosage of RYR preparations or comparing RYR with other herbal medicines were excluded.

### Validity assessment

The methodological quality of trials was assessed using the generation of the allocation sequence, the allocation concealment, double blinding, and withdrawals/dropouts [16-19].

### Data abstraction

One author (JL) extracted data and another author (JZ) cross-checked the data, and any disagreement was resolved by consensus. The following study characteristics were abstracted from the trials: design, participants and diagnosis, intervention regimen, and outcome measures.

### Data synthesis

We used the statistical package (RevMan 4.2) provided by the Cochrane Collaboration for data analyses. Dichotomous data were presented as relative risk (RR) and continuous outcomes as weighted mean difference (WMD), both with 95% confidence interval (CI). We assessed data by both random effects and fixed effect analyses, but reported the random effect analysis if the heterogeneity was significant, which was assessed by the *I*^2 ^statistic and used P < 0.10 as significance limit [20].

## Results

### Included trials

We identified 647 records on RYR preparations from electronic and manual searches. By reading titles and abstracts, we excluded 275 citations that were clearly duplicates, review articles, or non-clinical studies. A total of 372 articles published in Chinese or English were retrieved for further assessment. Of these, 279 articles were excluded because they were non-controlled clinical studies or randomized trials with different research objectives. Two of these were ongoing placebo-controlled trials testing 'Hypocol' in Norway and 'Lipolysar' in Italy [21], but data were not available while writing this report. In total, 93 randomized clinical trials [6-8,22-111] were identified and they reported to allocate participants with primary hyperlipidemia (n = 9625) randomly to RYR preparation or no treatment (2 trials), placebo (8 trials), statins (37 trials), other lipid-lowering agents (42 trials), or to a different RYR preparation (7 trials), in which three trials had more than two arms. The 93 trials were parallel group trials, and 91 were published in Chinese and two published in English [7,49]. Three RYR preparations were tested in the included trials: The RYR dietary supplement (Cholestin), and the Chinese proprietary medicines *Xuezhikang *and *Zhibituo*. Their constituents, dosages, and treatment regimens are listed in Table 1. All trials reported lipid profile outcome and 77 trials also reported adverse effects.

**Table 1:** Composition and treatment regimens of red yeast rice preparations

**Preparation**	**Composition**	**Dosage**	**Administration**
Red yeast rice dietary supplement (Cholestin) capsules	Extracts of fermented *Monascus purpureus *rice	2.4 g/day (containing 5 mg lovastatin)	0.6 g/cap 2 caps, twice daily
*Xuezhikang *capsule	Extracts from fermented Monascus *purpureus *rice, *Fructus Crataegi*, *Radix Salviae miltiorrhizae*, *Rhizoma Curcumae longae*, *Radix Rhizoma rhei*, etc.	1.2 g/day (containing 10 mg lovastatin)	0.3 g/cap 2 caps, twice daily
*Zhibituo *tablet	Extracts from fermented Monascus *purpureus *rice, *Fructus Crataegi*, *Rhizoma Atractylodis macrocephalae*, *Rhizoma Alismatis orientalis*, etc.	3.15 g/day (containing lovastatin 9 mg)	0.35 g/tab 3 tabs, thrice daily

### Methodological quality of included trials

Of the 93 trials, only three trials reported the methods to generate the allocation sequence (random number table or permuted blocks) [7,32,70], and two trials were assessed as having adequate concealment [7,29]. Five trials applied double-blinding [7,25-27,30], and three trials blinded the outcome assessors [29,51,106]. One trial reported prior sample size estimation and information on withdrawal/dropout [7], but no trial mentioned intention-to-treat analysis. Accordingly, the included trials had generally low methodological quality. All trials provided baseline data for the comparability among groups. The average sample size of the randomized trials was 103, ranging from 28 to 450 participants per trial.

### Total cholesterol (TC) levels (Tables 2 and 3)

**Table 2:** Net benefit of RYR preparations in lipid profile in placebo-controlled trials

**No. of subjects**		**Baseline**	**Post-treatment**	**Difference**		**P value**
					
		**Mean (SD)**	**Mean (SD)**	**Mean (95% CI)**	**% Change**	
**TC levels (mmol/L)**						
Cholestin	42	6.47 (0.78)	5.43 (0.80)	-1.04 (-1.38 to -0.70)	-16%	< 0.00001
Placebo	41	6.59 (0.75)	6.47 (0.93)	-0.12 (-0.49 to 0.25)	-2%	0.52
	30	6.68 (0.98)	5.31 (0.84)	-1.37 (-1.83 to -0.91)	-21%	
	33	5.81 (0.63)	4.26 (0.63)	-1.55 (-1.85 to -1.25)	-27%	
*Xuezhikang*	101	7.30 (1.40)	5.90 (1.40)	-1.40 (-1.79 to -1.01)	-19%	
	30	5.65 (1.31)	3.16 (1.31)	-2.49 (-3.15 to -1.83)	-44%	
	Subtotal: 194			-1.63 (-2.00 to -1.26)		< 0.00001
	28	6.72 (0.97)	6.43 (0.93)	-0.29 (-0.79 to 0.21)	-4%	
	30	5.84 (0.67)	4.94 (0.67)	-0.90 (-1.24 to -0.56)	-15%	
Placebo	51	6.80 (1.40)	6.70 (1.40)	-0.10 (-0.64 to 0.44)	-1%	
	20	5.55 (1.02)	4.95 (1.02)	-0.60 (-1.23 to 0.03)	-11%	
	Subtotal: 129			-0.50 (-0.90 to -0.11)		0.01
	9	5.90 (0.95)	4.94 (0.65)	-0.96 (-1.71 to -0.21)	-16%	
*Zhibituo*	30	6.70 (1.10)	5.80 (0.90)	-0.90 (-1.41 to -0.39)	-13%	
	104	7.10 (1.70)	5.60 (1.30)	-1.50 (-1.91 to -1.09)	-21%	
	Subtotal: 143			-1.17 (-1.59 to -0.74)		< 0.00001
	9	5.83 (0.57)	5.56 (0.53)	-0.27 (-0.78 to 0.24)	-5%	
Placebo	30	6.70 (1.50)	6.40 (1.30)	-0.30 (-1.01 to 0.41)	-4%	
	101	6.00 (1.80)	6.50 (0.70)	0.50 (0.12 to 0.88)	8%	
	Subtotal: 140			0.02 (-0.56 to 0.60)		0.95
						
**TG levels (mmol/L)**						
Cholestin	42	1.50 (0.54)	1.40 (0.50)	-0.10 (-0.32 to 0.12)	-7%	0.38
Placebo	41	1.61 (0.52)	1.65 (0.53)	0.04 (-0.19 to 0.27)	2%	
	30	2.84 (0.57)	2.38 (0.62)	-0.46 (-0.76 to -0.16)	-16%	
	33	2.10 (0.92)	1.82 (0.92)	-0.28 (-0.72 to 0.16)	-13%	
*Xuezhikang*	101	3.60 (2.40)	2.30 (1.60)	-1.30 (-1.86 to -0.74)	-36%	
	30	2.62 (0.58)	1.47 (0.58)	-1.15 (-1.44 to -0.86)	-44%	
	Subtotal: 194			-0.78 (-1.26 to -0.31)		0.001
	28	2.74 (0.73)	2.57 (0.69)	-0.17 (-0.54 to 0.20)	-6%	
Placebo	30	2.14 (0.94)	1.91 (0.94) 3.00 (1.60)	-0.23 (-0.71 to 0.25) -0.30 (-0.92 to 0.32)	-11%	
	51	3.30 (1.60)	3.00 (1.60)	-0.30 (-0.92 to 0.32)	-9%	
	20	2.50 (0.50)	2.11 (0.50)	-0.39 (-0.70 to -0.08)	-16%	
	Subtotal: 129			-0.29 (-0.49 to -0.09)		0.005
	13	2.46 (1.23)	1.79 (0.57)	-0.67 (-1.41 to 0.07)	-27%	
*Zhibituo*	30	3.40 (0.90)	2.10 (1.10)	-1.30 (-1.81 to -0.79)	-38%	
	104	3.40 (1.50)	2.30 (1.30)	-1.10 (-1.48 to -0.72)	-32%	
	Subtotal: 147			-1.10 (-1.38 to -0.82)		< 0.00001
	9	2.56 (0.88)	2.36 (0.64)	-0.20 (-0.91 to 0.51)	-8%	
	30	3.40 (1.10)	3.10 (1.20)	-0.30 (-0.88 to 0.28)	-9%	
Placebo	101	3.10 (0.40)	2.50 (1.40)	-0.60 (-0.88 to -0.32)	-19%	
	Subtotal: 140			-0.50 (-0.74 to -0.26)		< 0.0001
						
**LDL-C levels (mmol/L)**						
Cholestin	42	4.47 (0.70)	3.49 (0.70)	-0.98 (-1.28 to -0.68)	-22%	< 0.00001
Placebo	41	4.65 (0.78)	4.53 (0.85)	-0.12 (-0.47 to 0.23)	-3%	0.51
						
	30	3.94 (0.82)	2.83 (0.88)	-1.11 (-1.54 to -0.68)	-28%	
	33	4.13 (0.52)	2.80 (0.52)	-1.33 (-1.58 to -1.08)	-32%	
*Xuezhikang*	101	4.80 (1.60)	3.50 (1.40)	-1.30 (-1.71 to -0.89)	-27%	
	30	3.00 (1.03)	2.10 (1.03)	-0.90 (-1.42 to -0.38)	-30%	
	Subtotal: 194			-1.23 (-1.41 to -1.05)		<0.00001
	28	4.13 (0.94)	4.01 (0.86)	-0.12 (-0.59 to 0.35)	-3%	
	30	4.24 (0.53)	3.41 (0.53)	-0.83 (-1.10 to -0.56)	-20%	
Placebo	51	4.20 (1.60)	4.30 (1.60)	0.10 (-0.52 to 0.72)	2%	
	20	3.19 (0.87)	2.70 (0.87)	-0.49 (-1.03 to 0.05)	-15%	
	Subtotal: 129			-0.38 (-0.83 to 0.07)		0.10
*Zhibituo*	104	3.70 (1.60)	3.50 (1.40)	-0.20 (-0.61 to 0.21)	-5%	0.34
Placebo	101	3.70 (1.20)	3.70 (1.00)	0.00 (-0.30 to 0.30)	0%	1.00
						
**HDL-C levels (mmol/L)**						
Cholestin	42	1.29 (0.34)	1.29 (0.36)	0.00 (-0.15 to 0.15)	0%	1.00
Placebo	41	1.19 (0.26)	1.19 (0.28)	0.00 (-0.12 to 0.12)	0%	1.00
	30	1.50 (0.46)	1.67 (0.54)	0.17 (-0.08 to 0.42)	11%	
	33	1.37 (0.21)	1.16 (0.21)	-0.21 (-0.31 to -0.11)	-15%	
*Xuezhikang*	101	1.20 (0.40)	1.40 (0.30)	0.20 (0.10 to 0.30)	17%	
	30	1.20 (0.21)	1.22 (0.21)	0.02 (-0.09 to 0.13)	2%	
	Subtotal: 194			0.04 (-0.17 to 0.24)		0.73
	28	1.57 (0.69)	1.53 (0.32)	-0.04 (-0.32 to 0.24)	-3%	
	30	1.36 (0.24)	1.13 (0.24)	-0.23 (-0.35 to -0.11)	-17%	
	51	1.30 (0.40)	1.30 (0.30)	0.00 (-0.14 to 0.14)	0%	
Placebo	20	1.22 (0.25)	1.00 (0.25)	-0.22 (-0.37 to -0.07)	-18%	
	Subtotal: 129			-0.13 (-0.26 to -0.01)		0.04
	13	0.89 (0.41)	1.09 (0.41)	0.20 (-0.12 to 0.52)	22%	
	30	1.08 (0.11)	1.31 (0.17)	0.23 (0.16 to 0.30)	21%	
*Zhibituo*	104	0.85 (0.14)	0.98 (0.26)	0.13 (0.07 to 0.19)	15%	
	Subtotal: 147			0.18 (0.10 to 0.26)		< 0.0001
	13	0.92 (0.19)	0.90 (0.17)	-0.02 (-0.16 to 0.12)	-2%	
	30	0.99 (0.20)	1.02 (0.28)	0.03 (-0.09 to 0.15)	3%	
Placebo	101	0.89 (0.13)	0.80 (0.30)	-0.09 (-0.15 to -0.03)	-10%	
	Subtotal: 144			-0.04 (-0.12 to 0.03)		0.26

**Table 3:** Post-treatment total cholesterol levels (mmol/L) in randomized controlled trials

**Interventions**	**No. of trials [references]**	**No. of participants**	**Weighted mean difference (95% confidence interval)**	**P value**
**RYR vs. no intervention/placebo**				
*Zhibituo *vs. no intervention	2 [22, 23]	112	-1.27 (-1.50 to -1.05)	< 0.00001
RYR supplement vs. placebo	1 [7]	83	-1.04 (-1.41 to -0.67)	< 0.00001
*Xuezhikang *vs. placebo	4 [8, 24–26]	323	-1.04 (-1.46 to -0.62)*	< 0.00001
*Zhibituo *vs. placebo	3 [27–29]	283	-0.80 (-1.03 to -0.57)	< 0.00001
***RYR vs. statins***				
*Xuezhikang *vs. simvastatin	15 [6, 30–43]	1455	0.05 (-0.27 to 0.37)*	0.76
*Xuezhikang *vs. pravastatin	7 [44–50]	594	- 0.20 (- 0.47 to 0.06)*	0.14
*Xuezhikang *vs. lovastatin	3 [51–53]	174	-0.05 (-0.27 to 0.18)	0.69
*Xuezhikang *vs. atorvastatin	1 [54]	60	-0.16 (-0.58 to 0.26)	0.46
*Xuezhikang *vs. fluvastatin	1 [55]	118	0.48 (0.24 to 0.72)	0.0001
*Zhibituo *vs. simvastatin	9 [32, 56–63]	728	0.11 (-0.03 to 0.25)	0.14
*Zhibituo *vs. provastatin	1 [22]	62	0.05 (-0.20 to 0.30)	0.70
*Zhibituo *vs. lovastatin	1 [57]	45	-0.11 (-0.48 to 0.26)	0.56
***RYR vs. non-statin drugs***				
*Xuezhikang *vs. inositol nicotinate	7 [65–71]	624	-0.56 (-0.81 to -0.31)*	< 0.0001
*Xuezhikang *vs. fenofibrate	5 [32, 73–76]	337	-0.13 (-0.46 to 0.20)*	0.44
*Xuezhikang *vs. gemfibrozil	3 [77–79]	156	-0.43 (-1.52 to 0.65)*	0.43
*Xuezhikang *vs. fish oils	2 [80, 81]	116	-0.81 (-1.11 to -0.50)	< 0.00001
*Xuezhikang *vs. alginic sodium diester	1 [84]	60	-1.08 (-1.38 to -0.78)	< 0.00001
Xuezhikang vs. conjugated estrogens	1 [85]	44	-0.87 (-1.20 to -0.54)	< 0.00001
*Xuezhikang *vs. elastase	1 [86]	107	-0.10 (-0.49 to 0.29)	0.61
*Xuezhikang *vs. biphenalbid	1 [87]	64	0.12 (-0.31 to 0.55)	0.59
*Zhibituo *vs. inositol nicotinate	8 [88–95]	608	-0.73 (-1.13 to -0.33)*	0.0004
*Zhibituo *vs. fish oils	6 [97–102]	491	-0.76 (-1.04 to -0.49)*	< 0.00001
*Zhibituo *vs. fenofibrate	2 [32, 104]	248	0.31 (0.04 to 0.59)	0.02
***RYR versus RYR***				
*Xuezhikang *vs. *Zhibituo*	7 [32, 106–111]	701	-0.03 (-0.25 to 0.20)*	0.82

* Random effects model

**Figure 1:**
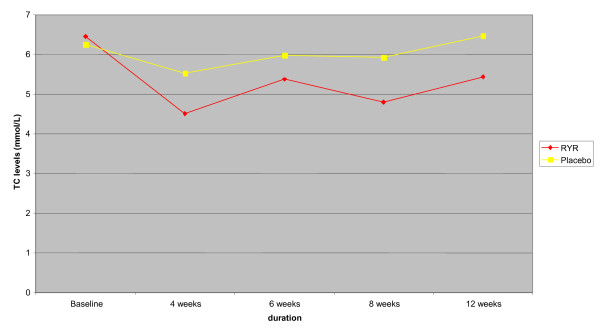
Total cholesterol levels during the treatment in 8 randomized placebo-controlled trials.

The three RYR preparations significantly reduced serum TC levels and the effect was reached at four weeks after the treatment and remained stable until 12 weeks (Figure 1). The percentage of TC level reduction was 16% for cholestin, 19%–44% for *Xuezhikang*, and 13%–21% for *Zhibituo *(Table 2). Compared with no treatment, *Zhibituo *showed a reduction of serum TC levels (WMD -1.27 mmol/L; 95% CI -1.50 to -1.05; 2 trials, n = 112) [22,23]. Compared with placebo, significant reduction of serum TC levels was found for Cholestin (WMD -1.04 mmol/L; 95% CI -1.41 to -0.67; 1 trial, n = 83) [7], *Xuezhikang *(WMD -1.04 mmol/L; 95% CI -1.46 to -0.62; 4 trials, n = 323) [8,24-26], and *Zhibituo *(WMD -0.80 mmol/L; 95% CI -1.03 to -0.57; 3 trials, n = 283) [27-29]. There was no significant heterogeneity among the trials (*I*^2 ^statistic test) (Table 3). Different treatment duration showed similar effect of RYR preparations in reducing TC levels compared with placebo by 4 weeks (WMD -0.96 mmol/L; 95% CI -1.49 to -0.43; 2 trials, n = 113) [25,26], 6 weeks (WMD -0.61; 95% CI -1.0 to -0.22; 2 trials, n = 78) [27,28], 8 weeks (WMD -1.06; 95% CI -1.39 to -0.73; 5 trials, n = 406) [7,8,24-26], and 12 weeks (WMD -1.04; 95% CI -1.41 to -0.67; 1 trial, n = 83) [7].

*Xuezhikang *and *Zhibituo *were compared with simvastatin, pravastatin, lovastatin, atorvastatin, or fluvastatin in 37 trials. There was no statistically significant difference in TC levels between RYR preparation and statins except for one trial, in which *Xuezhikang *was less effective than fluvastatin (WMD 0.48 mmol/L; 95% CI 0.24 to 0.72; n = 118) [55]. One trial presented data as number of subjects with at least 10% reduction of TC levels, and it showed no difference between *Xuezhikang *and lovastatin (64/69 vs. 68/76; RR 1.04; 95% CI 0.94 to 1.15) [64]. In these trials, *Xuezhikang *was used at dosage of 1.2 g/day (containing 10 mg of lovastatin), *Zhibituo *at 3.15 g/day (containing 9 mg of lovastatin), simvastatin at 10–20 mg/day, pravastatin at 10 mg/day, lovastatin at 20 mg/day, atorvastatin 10 mg/day, and fluvastatin 20 mg/day.

Compared with non-statin lipid-lowering agents, *Xuezhikang *was more effective in lowering TC levels than inositol nicotinate (WMD -0.56 mmol/L; 95% CI -0.81 to -0.31; 7 trials, n = 624) [65-71], fish oils (WMD -0.81 mmol/L; 95% CI -1.11 to -0.50; 2 trials, n = 116) [80,81]], alginic sodium diester (WMD -1.08 mmol/L; 95% CI -1.38 to -0.78; 1 trial, n = 60) [84], and conjugated estrogens (WMD -0.87 mmol/L; 95% CI -1.20 to -0.54; 1 trial, n = 44) in postmenopausal women[85]. More participants had 10% reduction of TC levels after treatment of *Xuezhikang *against inositol nicotinate (16/18 vs. 7/17; RR 2.16; 95% CI 1.20 to 3.90) [72]. *Xuezhikang *was better than fish oils in terms of more participants with 10% reduction of TC levels (WMD 1.36; 95% CI 1.14 to 1.63; 2 trials, n = 146) [82,83]. No significant difference was found between *Xuezhikang *and fenofibrate or gemfibrozil, *Xuezhikang *and elastase or biphenalbid in lowering TC levels. However, *Zhibituo *was less effective than fenofibrate (WMD 0.31 mmol/L; 95% CI 0.04 to 0.59; 2 trials, n = 248) [32,104], but more effective than inositol nicotinate (WMD -0.73 mmol/L; 95% CI -1.13 to -0.33; 8 trials, n = 608) [88-95], and fish oils (WMD -0.76 mmol/L; 95% CI -1.04 to -0.49; 6 trials, n = 491) [97-102] by random effect model due to significant heterogeneity (Table 3). More participants treated by *Zhibituo *had 10% reduction of TC levels compared with those treated by inositol nicotinate (23/30 vs. 13/30; RR 1.77; 95% CI 1.12 to 2.79) [96] or those treated by fish oils (18/25 vs. 7/25; RR 2.57; 95% CI 1.31 to 5.05) [103]. *Zhibituo *appeared superior to alginic sodium diester for the number of participants with 10% reduction of TC levels (105/121 vs. 67/89; RR 1.15; 95% CI 1.00 to 1.32) [105].

*Xuezhikang *did not differ from *Zhibituo *in TC-lowering effect (WMD -0.03 mmol/L; 95% CI -0.25 to 0.20; 7 trials, n = 701) [32,106-111] (Table 3).

### Triglycerides (TG) levels (Tables 2 and 4)

**Table 4:** Post-treatment triglycerides levels (mmol/L) in randomized controlled trials

**Interventions**	**No. of trials [references]**	**No. of participants**	**Weighted mean difference (95% confidence interval)**	**P value**
**RYR vs. no intervention/placebo**				
*Zhibituo *vs. no intervention	2 [22, 23]	112	-0.54 (-0.77 to -0.32)	< 0.00001
RYR supplement vs. placebo	1 [7]	83	-0.25 (-0.47 to -0.03)	0.03
*Xuezhikang *vs. placebo	4 [8, 24–26]	323	-0.40 (-0.70 to -0.10)*	0.008
*Zhibituo *vs. placebo	3 [27–29]	291	-0.55 (-0.99 to -0.10)	0.02
**RYR vs. statins**				
*Xuezhikang *vs. simvastatin	14 [6, 30–42]	1251	-0.08 (-0.25 to 0.10)*	0.39
*Xuezhikang *vs. pravastatin	7 [44–50]	592	0.04 (- 0.29 to 0.38)*	0.79
*Xuezhikang *vs. lovastatin	3 [51–53]	168	-0.07 (-0.16 to 0.01)	0.09
*Xuezhikang *vs. atorvastatin	1 [54]	60	-0.02 (-0.16 to 0.12)	0.78
*Xuezhikang *vs. fluvastatin	1 [55]	118	0.09 (-0.1 4 to 0.32)	0.44
*Zhibituo *vs. simvastatin	9 [32, 56–63]	732	0.05 (-0.17 to 0.26)*	0.67
*Zhibituo *vs. provastatin	1 [22]	62	-0.02 (-0.19 to 0.15)	0.81
*Zhibituo *vs. lovastatin	1 [57]	45	-0.21 (- 0.61 to 0.19)	0.30
**RYR vs. non-statin drugs**				
*Xuezhikang vs*. inositol nicotinate	7 [65–71]	636	-0.06 (-0.20 to 0.08)	0.38
*Xuezhikang *vs. fenofibrate	5 [32, 73–76]	337	0.42 (-0.17 to 1.01)*	0.16
*Xuezhikang *vs. gemfibrozil	3 [77–79]	160	0.41 (0.30 to 0.51)	< 0.00001
*Xuezhikang *vs. fish oils	2 [80, 81]	112	-0.71 (-0.97 to -0.44)	< 0.00001
*Xuezhikang *vs. alginic sodium diester	1 [84]	60	0.04 (-0.21 to 0.29)	0.75
*Xuezhikang *vs.conjugated estrogens	1 [85]	44	-0.82 (-1.31 to -0.33)	0.001
*Xuezhikang *vs. elastase	1 [86]	107	0.00 (-0.31 to 0.31)	1.00
*Xuezhikang *vs. biphenalbid	1 [87]	64	-0.43 (-0.8 1 to -0.05)	0.03
*Zhibituo *vs. inositol nicotinate	7 [88–93, 95]	598	-0.39 (-0.62 to -0.16)*	0.0008
*Zhibituo *vs. fish oils	5 [97–100, 102]	394	-0.12 (-0.29 to 0.05)	0.17
*Zhibituo *vs. fenofibrate	2 [32, 104]	248	0.33 (-0.12 to 0.78)	0.15
**RYR vs. RTR**				
*Xuezhikang *vs. *Zhibituo*	7 [32, 106–111]	727	0.05 (-0.17 to 0.27)*	0.66

* Random effects model

There was a 13%–44% reduction of TG levels after treatment *by Xuezhikang*, 27%–38% by *Zhibituo*, and 7% by Cholestin. Compared with no treatment, *Zhibituo *showed a significant effect on reducing TG levels (WMD -0.54 mmol/L; 95% CI -0.77 to -0.32; 2 trials, n = 112) [22,23]. Compared with placebo, all three RYR preparations significantly reduced TG levels (Cholestin: WMD -0.25 mmol/L; 95% CI -0.47 to -0.03; 1 trial, n = 83 [7]; *Xuezhikang*: WMD -0.40 mmol/L; 95% CI -0.70 to -0.10; 4 trials, n = 323 [8,24-26]; and *Zhibituo*: WMD -0.55 mmol/L; 95% CI -0.99 to -0.10; 3 trials, n = 283) [27-29] (Table 4). Different treatment duration showed similar effect of RYR preparations in reducing TG levels compared with placebo by 4 weeks (WMD -0.32 mmol/L; 95% CI -0.58 to -0.07; 2 trials, n = 113) [25,26], 6 weeks (WMD -0.74; 95% CI -1.10 to -0.37; 2 trials, n = 86) [27,28], 8 weeks (WMD -0.35; 95% CI -0.5 to -0.21; 5 trials, n = 406) [7,8,24-26], and 12 weeks (WMD -0.25; 95% CI -0.47 to -0.03; 1 trial, n = 83) [7].

There was no statistically significant difference in TG levels after treatment between *Xuezhikang *or *Zhibituo *and simvastatin, pravastatin, lovastatin, atorvastatin, or fluvastatin. One trial presented data as number of subjects with at least 20% reduction of TG levels, and it showed no difference between *Xuezhikang *and lovastatin (47/60 vs. 59/77; RR 1.02; 95% CI 0.85 to 1.23) [64].

Compared with non-statin lipid lowering agents, there was no significant difference between *Xuezhikang *and inositol nicotinate, fenofibrate, alginic sodium diester, or elastase for TG levels. There was no difference between *Xuezhikang *and inositol nicotinate in number of participants with over 20% reduction of TG levels (11/16 vs. 10/16; RR 1.10; 95% CI 0.67 to 1.82) [72], and between *Xuezhikang *and fish oils (58/78 vs. 44/70; RR 1.15; 95% CI 0.8 to 1.64) [82,83]. However, *Xuezhikang *was less effective than gemfibrozil (WMD 0.41 mmol/L; 95% CI 0.30 to 0.51; 3 trials, n = 160) [77-79], but better than fish oils (WMD -0.71 mmol/L; 95% CI -0.97 to -0.44; 2 trials, n = 112) [80,81], conjugated estrogens (WMD -0.82 mmol/L; 95% CI -1.31 to -0.33; 1 trial, n = 44) [85] in postmenopausal women, and biphenalbid (WMD -0.43 mmol/L; 95% CI -0.81 to -0.05; 1 trial, n = 64) [87]. *Zhibituo *showed a significant better TG-lowering effect (WMD -0.39 mmol/L; 95% CI -0.62 to -0.16; 7 trials, n = 598) [88-93,95] compared with inositol nicotinate. However, there was no significant difference between *Zhibituo *and inositol nicotinate in the number of participants with over 20% reduction of TG levels (9/30 vs. 4/30) in one trial [96]. *Zhibituo *did not differ from fish oils (WMD -0.12 mmol/L; 95% CI -0.29 to 0.05; 5 trials, n = 394) [97-100,102] or fenofibrate (WMD 0.33 mmol/L; 95% CI -0.12 to 0.78; 2 trials, n = 248) [32,104] (Table 4). In a small trial more participants appeared to have a 20% reduction of TG levels by *Zhibituo *than by fish oils (20/23 vs. 9/19; RR 1.84; 95% CI 1.11 to 3.03) [103]. There was a marginal effect of *Zhibituo *compared with alginic sodium diester for the number of participants with over 20% reduction of TG levels (69/121 vs. 38/89; RR 1.34; 95% CI 1.00 to 1.78) [105].

There was no significant difference between *Xuezhikang *and *Zhibituo *in reducing TG levels (WMD 0.05 mmol/L; 95% CI -0.17 to 0.27; 7 trials, n = 727) (Table 4).

### Low density lipoprotein cholesterol (LDL-C) levels (Tables 2 and 5)

**Table 5:** Post-treatment low-density lipoprotein cholesterol levels (mmol/L) in randomized controlled trials

**Interventions**	**No. of trials [references]**	**No. of participants**	**Weighted mean difference (95% confidence interval)**	**P value**
**RYR vs. no intervention/placebo**				
*Zhibituo *vs. no intervention	1 [22]	62	-0.16 (-0.71 to 0.39)	0.57
RYR supplement vs. placebo	1 [7]	83	-1.04 (-1.38 to -0.70)	< 0.00001
*Xuezhikang *vs. placebo	4 [8, 24–26]	323	-0.74 (-0.93 to -0.55)	< 0.00001
*Zhibituo *vs. placebo	1 [29]	205	-0.20 (-0.53 to 0.13)	0.24
**RYR vs. statins**				
*Xuezhikang *vs. simvastatin	13 [6, 30–34, 36–38, 40–43]	1238	0.14 (-0.05 to 0.33)*	0.14
*Xuezhikang *vs. pravastatin	7 [44–50]	587	-0.09 (- 0.20 to 0.02)*	0.11
*Xuezhikang *vs. lovastatin	3 [51–53]	191	0.00 (- 0.26 to 0.27)	0.98
*Xuezhikang *vs. atorvastatin	1 [54]	60	0.20 (-0.10 to 0.50)	0.19
*Xuezhikang *vs. fluvastatin	1 [55]	118	0.14 (-0.10 to 0.38)	0.26
*Zhibituo *vs. simvastatin	8 [32, 56–62]	601	0.22 (0.04 to 0.39)	0.02
*Zhibituo *vs. provastatin	1 [22]	62	-0.11 (-0.60 to 0.38)	0.66
*Zhibituo *vs. lovastatin	1 [57]	45	0.03 (-0.30 to 0.36)	0.86
**RYR vs. non-statin drugs**				
Xuezhikang vs. inositol nicotinate	4 [66–68, 70]	299	-0.63 (-0.96 to -0.30)	0.0002
*Xuezhikang *vs. fenofibrate	3 [32, 74, 76]	220	-0.10 (-1.05 to 0.85)*	0.84
*Xuezhikang *vs. gemfibrozil	3 [77–79]	152	-0.34 (-0.58 to -0.10)	0.005
*Xuezhikang *vs. fish oils	1 [81]	95	-0.89 (-1.41 to -0.37)	0.0008
*Xuezhikang *vs. conjugated estrogens	1 [85]	44	-0.10 (-0.43 to 0.23)	0.55
*Xuezhikang *vs. biphenalbid	1 [87]	64	-0.06 (-0.32 to 0.20)	0.65
*Zhibituo *vs. fish oils	5 [97–101]	489	-0.57 (-0.70 to -0.45)	< 0.00001
*Zhibituo *vs. fenofibrate	1 [32]	90	0.3 1 (0.04 to 0.58)	0.02
**RYR vs. RYR**				
*Xuezhikang *vs. *Zhibituo*	5 [32, 106, 108, 109, 111]	628	-0.08 (-0.18 to 0.02)	0.12

* Random effects model

There was a 22% reduction of LDL-C levels by Cholestin and 27%–32% by *Xuezhikang*, but 5% by *Zhibituo. Zhibituo *appeared to have no effect on reducing LDL-C levels compared with no treatment [22] or placebo [28]. The relative benefit of reducing LDL-C levels by Cholestin against placebo was WMD -1.04 mmol/L (95% CI -1.38 to -0.70; 1 trial, n = 83) [7], and by *Xuezhikang *against placebo (WMD) -0.74 mmol/L; 95% CI -0.93 to -0.55; 4 trials, n = 323) [8,23-25] (Table 5). Different treatment duration showed similar effect of RYR preparations in reducing LDL-C levels compared with placebo by 4 weeks (WMD -0.77 mmol/L; 95% CI -1.0 to -0.54; 2 trials, n = 113) [25,26], 8 weeks (WMD -0.87; 95% CI -1.15 to -0.60; 5 trials, n = 406) [7,8,24-26], and 12 weeks (WMD -1.04; 95% CI -1.38 to -0.70; 1 trial, n = 83) [7].

*Xuezhikang *did not differ from simvastatin, pravastatin, lovastatin, atorvastatin or fluvastatin for post-treatment LDL-C levels. *Zhibituo *appeared to have the same effect as pravastatin or lovastatin, but was less effective than simvastatin (WMD 0.22 mmol/L; 95% CI 0.04 to 0.39; 8 trials, n = 601) [32,56-62]. Compared with non-statin lipid-lowering agents, *Xuezhikang *was similar to fenofibrate, conjugated estrogens or biphenalbid, but significantly better in reducing LDL-C levels than inositol nicotinate (WMD -0.63 mmol/L; 95% CI -0.96 to -0.30; 4 trials, n = 299) [66-68,70], gemfibrozil (WMD -0.34 mmol/L; 95% CI -0.58 to -0.10; 3 trials, n = 152) [77-79], and fish oils (WMD -0.89 mmol/L; 95% CI -1.41 to -0.37, 1 trial, n = 95) [81]. *Zhibituo *was better than fish oils in reducing LDL-C levels (WMD -0.57 mmol/L; 95% CI -0.70 to -0.45; 5 trials, n = 489) [97-101], but less effective than fenofibrate (WMD 0.31 mmol/L; 95% CI 0.04 to 0.58; 1 trial, n = 90) [32] (Table 5).

No significant difference was found between *Xuezhikang *and *Zhibituo *in LDL-C levels (-0.08 mmol/L;-0.18 to 0.02; 5 trials, n = 628) [32,106,108,109,111] (Table 5).

### High density lipoprotein cholesterol (HDL-C) levels (Tables 2 and 6)

**Table 6:** Post-treatment high-density lipoprotein cholesterol levels (mmol/L) in randomized controlled trials

**Interventions**	**No. of trials [references]**	**No. of participants**	**Weighted mean difference its (95% confidence interval)**	**P value**
**RYR vs. no intervention/placebo**				
*Zhibituo *vs. no intervention	1 [22]	62	0.21 (0.04 to 0.38)	0.02
RYR supplement vs. placebo	1 [7]	83	0.10 (-0.04 to 0.24)	0.16
*Xuezhikang vs*. placebo	4 [8, 24–26]	323	0.11 (0.05 to 0.17)	0.0008
*Zhibituo *vs. placebo	3 [27–29]	291	0.21 (0.15 to 0.27)	< 0.00001
**RYR vs. statins**				
*Xuezhikang *vs. simvastatin	14 [6, 30–34, 36–43]	1277	0.06 (-0.11 to 0.22)*	0.49
*Xuezhikang *vs. pravastatin	7 [44–50]	587	-0.01 (-0.06 to 0.03)	0.51
*Xuezhikang *vs. lovastatin	3 [51–53]	152	0.06 (0.00 to 0.11)	0.05
*Xuezhikang *vs. atorvastatin	1 [54]	60	0.01 (-0.17 to 0.19)	0.91
*Xuezhikang *vs. fluvastatin	1 [55]	118	-0.02 (-0.1 0 to 0.06)	0.62
*Zhibituo *vs. simvastatin	9 [32, 56–63]	666	-0.07 (-0.12 to -0.03)	0.0009
*Zhibituo *vs. provastatin	1 [22]	62	-0.02 (-0.22 to 0.18)	0.85
*Zhibituo *vs. lovastatin	1 [57]	45	-0.07 (-0.23 to 0.09)	0.39
**RYR vs. non-statin drugs**				
*Xuezhikang *vs. inositol nicotinate	7 [65–71]	608	0.17 (0.06 to 0.28)*	0.002
*Xuezhikang *vs. fenofibrate	4 [32, 73, 74, 76]	257	0.03 (-0.06 to 0.13)	0.49
Xuezhikang vs. gemfibrozil	2 [77, 79]	108	-0.03 (-0.3 5 to 0.28)	0.83
*Xuezhikang *vs. fish oils	2 [80, 81]	70	0.17 (0.09 to 0.25)	< 0.0001
*Xuezhikang *vs. alginic sodium diester	1 [84]	60	0.86 (0.75 to 0.97)	< 0.00001
*Xuezhikang *vs. conjugated estrogens	1 [85]	44	0.00 (-0.09 to 0.09)	1.00
Xuezhikang vs. elastase	1 [86]	107	0.20 (0.10 to 0.30)	< 0.0001
*Xuezhikang *vs. biphenalbid	1 [87]	64	0.25 (0.11 to 0.39)	0.0003
*Zhibituo *vs. inositol nicotinate	6 [89–91, 93–95]	422	0.18 (0.09 to 0.27)*	< 0.0001
*Zhibituo *vs. fish oils	6 [97–102]	400	0.14 (0.06 to 0.23)*	0.001
*Zhibituo *vs. fenofibrate	2 [32, 104]	248	-0.13 (-0.37 to 0.11)	0.28
**RYR vs. RYR**				
*Xuezhikang *vs. *Zhibituo*	7 [32, 106–111]	627	0.04 (-0.02 to 0.11)*	0.20

* Random effects model

There was an increase of HDL-C levels between 15% and 22% by *Zhibituo*. However, the findings for *Xuezhikang *were not consistent ranging from a 2% to 17% increase and 15% decrease in four trials. Cholestin did not change the HDL-C levels after the treatment [7]. A beneficial effect of increasing HDL-C levels was shown when *Xuezhikang *was compared with placebo (WMD 0.11 mmol/L; 95%CI 0.05 to 0.17; 4 trials, n = 323) [8,24-26], and when *Zhibituo *was compared with no treatment (WMD 0.21 mmol/L; 95% CI 0.04 to 0.38; 1 trial, n = 62) [22] and with placebo (WMD 0.21 mmol/L; 95% CI 0.15 to 0.27; 3 trials, n = 291) [27-29] (Table 6). Different treatment durations showed that of RYR preparations increased HDL-C levels compared with placebo by 6 weeks (WMD 0.27; 95% CI 0.17 to 0.38; 2 trials, n = 86) [27,28] and 8 weeks (WMD 0.11; 95% CI 0.05 to 0.16; 5 trials, n = 406) [7,8,24-26]. There was no significant difference between RYR and placebo at 4 weeks and at 12 weeks for HDL-C levels [7,25,26].

Compared with statins, *Xuezhikang *appeared better than lovastatin in raising HDL-C levels (WMD 0.06 mmol/L; 95% CI 0.00 to 0.11; 3 trials, n = 152) [51,53]. *Zhibituo *was inferior to simvastatin (WMD -0.07 mmol/L; 95% CI -0.12 to -0.03; 9 trials, n = 666) [32,56-63]. There was no significant difference among other comparisons of RYR preparations and statins. Compared with non-statins, *Xuezhikang *was superior to inositol nicotinate (WMD 0.17 mmol/L; 95% CI 0.06 to 0.28; 7 trials, n = 608) by random effects model [65-71], fish oils (WMD 0.17 mmol/L; 95% CI 0.09 to 0.25; 2 trials, n = 70) [80,81], alginic sodium diester (WMD 0.86 mmol/L; 95% CI 0.75 to 0.97; 1 trial, n = 60) [84], elastase (WMD 0.20 mmol/L; 95% CI 0.10 to 0.30; 1 trial, n = 107) [86], and to biphenalbid (WMD 0.25 mmol/L; 95% CI 0.11 to 0.39; 1 trial, n = 64) [87]. There was no significant difference between *Xuezhikang *and fenofibrate, gemfibrozil, or estrogens in affecting HDL-C levels. *Zhibituo *was superior to inositol nicotinate (WMD 0.18 mmol/L; 95% CI 0.09 to 0.27; 6 trials, n = 422) [89-91,93-95] and to fish oils (WMD 0.14 mmol/L; 95% CI 0.06 to 0.23; 6 trials, n = 400) [97-102] both in random effects model. There was no significant difference between *Zhibituo *and fenofibrate (WMD -0.13 mmol/L; 95% CI -0.37 to 0.11; 2 trials, n = 248) [32,104].

No significant difference was found between *Xuezhikang *and *Zhibituo *in affecting HDL-C levels (WMD 0.04 mmol/L; 95% CI -0.02 to 0.11; 7 trials, n = 627) [32,106-111] (Table 6).

### Adverse effects

Seventy-seven trials reported outcomes of adverse effects, and the incidence rate ranged from 1.3% to 36%. The most commonly reported adverse effects were dizziness, low appetite, nausea, stomach-ache, abdominal distension, and diarrhoea. A small proportion of participants suffered from increased serum BUN and ALT levels. The trials did not report serious adverse events.

### Cost-effectiveness

One trial evaluated cost-effectiveness of *Xuezhikang *vs. pravastatin for treatment of hypercholesterolemia [49]. For a reduction of 1 mmol/L TC level, the cost of *Xuezhikang *and pravastatin was 57 USD and 78 USD respectively. For a reduction of 1 mmol/L TG level, the cost of *Xuezhikang *and pravastatin was 242 USD and 820 USD respectively; and for a reduction of 1 mmol/L LDL-C level, the cost was 59 USD and 84 USD respectively.

## Discussion

Based on this review and meta-analysis, three different kinds of RYR preparations tested by in randomized trials demonstrate beneficial effects on reducing TC, TG, and LDL-C levels, and on increasing HDL-C levels in individuals with hyperlipidemia. The treatment duration of RYR ranged from 4 to 24 weeks (median of 8 weeks), and the lipid modification effects have been shown at four weeks of the treatment, and the effects remained at 24 weeks of the treatment. Long-term follow-up effects after the treatment have not been reported by the trials. The use of RYR preparations seems safe and well tolerated.

Before accepting the findings of this review to form a basis for clinical practice, we need to consider the following weaknesses in this review. First, the randomized trials in this review had several methodological flaws in terms of insufficient reporting of generation methods of the allocation sequence, allocation concealment, and double blinding. The trials provided limited descriptions of study design, and most trials stated only that patients were randomly assigned; thus the information does not allow a judgement of whether or not it was conducted properly. We therefore state that the differences between RYR preparation and control drugs may be associated with the methodologically less rigorous trials [16-19]. The sample size for trials comparing RYR with statins or other established treatments was not justified and we do not know if the trials were designed as 'equivalence trials'. The limited number of trials with adequate quality prohibits us from performing meaningful sensitivity analyses to illuminate robustness of the results in the review.

Second, Vickers and colleagues [112] found that some countries, including China, publish unusually high proportions of positive results, for which publication bias is a possible explanation. All identified studies for this systematic review originated from China except one trial conducted in the USA and published in an international peer-reviewed journal [7]. Inability to identify unpublished eligible trials from the searching, trials with small samples and positive findings may raise the issue of publication bias.

There are some variations in RYR preparations and treatment regimens including composition, dosage and duration. Cholestin is an extract from RYR containing a special strain of yeast which produces monacolin K (lovastatin) [7]. *Xuezhikang *and *Zhibituo *are two Chinese proprietary medicines that contain other herbs in addition to RYR as main components. In some trials, placebo effects are substantial compared with baseline as demonstrated in trials of *Xuezhikang *where placebo treatment achieved 1% to 15% reduction of TC levels, and 6% to 16% reduction of TG levels (Table 2). Therefore, in non-placebo-controlled and non-double blind trials, placebo effects may add to the complexity of interpreting the present findings of the overall beneficial effects, and the interpretation should be taken with caution.

Given the generally low methodological quality of the randomized trials and potential publication bias, we suggest further rigorously designed trials are still needed before RYR preparation could be recommended for clinical use or as an alternative treatment to statins. The currently ongoing placebo-controlled trials in Europe may provide useful information [21]. In addition to anti-hyperlipidemic effects of RYR preparations, cost-effectiveness and safety should be further investigated in future trials [113].

## Conclusion

Current evidence from randomized trials shows short-term beneficial effects of RYR preparations on lipid modification. More rigorous trials are needed, and long-term effects and safety should be investigated if RYR preparations are to be recommended as one of the alternative treatments for primary hyperlipidemia.

## Competing interests

The author(s) declare that they have no competing interests.

## Authors' contributions

JL conceived, designed, drafted the review, and performed study selection, data extraction, analyses, and interpretation. JZ did the literature search, study selection, and cross-checked the data extraction; YS developed the search strategy, performed electronic searches and retrieved articles; SG, TA, and VF provided methodological perspectives, and revised the review. All authors contributed to the writing of the review.
